# PSOGI consensus on minimally invasive surgery for peritoneal surface malignancy

**DOI:** 10.1093/bjs/znag019

**Published:** 2026-03-04

**Authors:** Alvaro Arjona-Sanchez, Aditi Bhatt, Mufaddal Kazi, S P Somashekar, Mustafa Raoof, Delia Cortés, Shigeki Kusamura, Kurt Van der Speeten, Antonio Sommariva, Mohammad Alyami, Olivier Glehen, PSOGI Collaborators, Alexander Heriot, Almog Ben-Yaacov, Andrea Di Giorgio, Andreas Brandl, Andrew M Lowy, Armando Sardi, Avanish Saklani, Beate Rau, Brian Badgwell, Claramae Chia, Dario Baratti, Dileep Damodaran, Edward Levine, Emel Canbay, Clarisse Eveno, Faheez Mohamed, Fernando Pereira, Gabriel Glockzin, Diane Goere, Ignace De Hingh, Jeremiah Deneve, Jesus Esquivel, Jula Veerapong, Konstantinos Votanopoulos, Lana Bijelic, Laura Lambert, Lucas Sideris, Luis Gonazalez Bayon, M Haroon A Choudry, Marcello Guaglio, Frederich Marchal, Martin Hübner, Michele De Simone, Nabila Ansari, Nayef Alzahrani, Niels Kok, Oliver Eng, Olivia Sgarbura, Paolo Sammartino, Pedro Antonio Cascales Campos, Pedro Bretcha-Boix, Peter Cashin, Marc Pocard, Pompiliu Piso, Ramakrishnan Ayaloor Seshadri, Robert M Barone, Roman Yarema, Ruiqing Ma, Sanjeev Dayal, Sanket Mehta, Santiago González-Moreno, Satish Warrier, Sean Dineen, Selman Sokmen, Sherif Abdel-Misih, Spiliotis John, Abdelkader Taibi, Tom Cecil, Vadim Gushchin, Victor Verwaal, Willemien van Driel, Wim Ceelen, Yan Li, Yanghee Woo, Yonemura Yutaka, Zoltan Novak, Naoual Bakrin, Joel Baumgartner, Cecile Brigand, Jeremiah Deneve, Marcello Deraco, Pierre Dube, Vahan Kepenekian, Brian Loggie, Andrew Lowy, Brendan Moran, David Morris, Claudio Quadros, Francois Quenet, Paul Sugarbaker, Antonio Apostolos Tentes, Melissa Teo, Kiran Turaga, Snita Sinukumar, Marc Lopez, Amine Souadka, Rafael Seitenfuis

**Affiliations:** Unit of Oncological and Pancreatic Surgery, University Hospital Reina Sofía and Maimónides Biomedical Research Institute of Córdoba (IMIBIC)/Reina Sofia University Hospital/University of Córdoba, Cordoba, Spain; Department of Surgical Oncology, Shalby Cancer and Research Institute, Ahmedabad, India; Department of Surgical Oncology, Tata Memorial Hospital, Homi Bhabha National Institute, Mumbai, India; Department of Surgical Oncology, Aster International Institute of Oncology, Bangalore, India; Department of Surgery, City of Hope National Medical Center, Duarte, California, USA; Department of Surgical Oncology, University Hospital Viamed Santa Elena (IVOQA), Madrid, Spain; Department of Surgical Oncology, Fondazione IRCCS Istituto Nazionale dei Tumori, Milan, Italy; Department of Surgical Oncology, Zeikenhuis Oost-Limberg, Genk, Belgium; Department of Surgical Oncology, Veneto Institute of Oncology IOV-IRCCS, Padua, Italy; Department of Surgery, Oncology Centre, King Khalid Hospital, Najran, Saudi Arabia; Department of Surgical Oncology, Centre Hospitalier Lyon-sud, Lyon, France; CICLY, Lyon 1 University, Lyon, France

## Introduction

Cytoreductive surgery (CRS) + hyperthermic intraperitoneal chemotherapy (HIPEC) is an effective strategy for the treatment of patients with peritoneal malignancy (PM). However, it continues to be considered major surgery, with high morbidity and mortality rates, which should only be performed in reference centres. Minimally invasive surgery (MIS) is an emerging tool in the field of PM, improving diagnosis and staging, as well as allowing CRS + HIPEC to be performed with improved perioperative outcomes^1,2^.

Performing CRS using a minimally invasive (MI) approach is challenging, especially when operating in the upper abdomen, porta hepatis, and lesser sac. Additional difficulties that can be encountered during MIS include adhesions caused by the peritoneal spread of a tumour or prior surgery, as well as a lack of tactile sensation, which is important for identifying all disease sites correctly^3^. With increasing awareness and improvements in imaging techniques, PM is now frequently diagnosed at an early stage when the disease is limited to a few abdominal regions. Earlier diagnosis has led several surgeons to explore the role of MIS in patients with a low peritoneal cancer index (PCI). Although there is a lack of strong evidence, the published studies showed shorter recovery times without compromising oncological outcomes^1,2,4–6^.

The three potential scenarios in which MIS could be implemented in the field of PM are: staging laparoscopy (SL), risk-reducing (RR) MI CRS + HIPEC, and MI CRS + HIPEC. The aim of this consensus was to address questions regarding indications for these approaches and optimal techniques for performing them.

## Methods

This consensus was organized by the Peritoneal Surface Oncology Group International (PSOGI) using a modified Delphi technique^7^ due to heterogeneous views, lack of strong evidence, and the need for structured expert agreement. Surgical oncologists with significant experience in performing CRS and MIS were invited to join the working group and a steering group of 11 members was formed.

The consensus was divided into four important topics: SL, RR MI CRS + HIPEC, MI CRS + HIPEC, and limitations of MIS. SL was defined as a laparoscopic procedure to describe the PCI and/or establish resectability, RR MI CRS + HIPEC was defined as MI (laparoscopic or robotic) CRS + HIPEC performed in patients with a primary tumour known to carry a high risk of peritoneal disease (locally advanced gastric and colon cancer) or low-grade appendiceal mucinous neoplasm (LAMN) type II^8^, and MI CRS + HIPEC was defined as MI (laparoscopic or robotic) CRS + HIPEC performed in patients with identifiable PM. Low-grade tumours included low-grade mucinous carcinoma peritonei (LGPMP), multicystic mesothelioma, and well differentiated papillary mesothelioma. High-grade tumours included all invasive carcinomas and high-grade mucinous carcinoma peritonei (HGMCP).

### Developing the questionnaire

A literature search was performed for each of the four main topics in the MEDLINE and Embase databases using the following keywords: ‘cytoreductive surgery’, ‘minimally-invasive cytoreductive surgery’, ‘laparoscopic cytoreductive surgery’, ‘risk-reducing cytoreductive surgery’, ‘prophylactic HIPEC’, ‘peritoneal malignancy’, and ‘staging laparoscopy’. Manuscripts published in the English language from 1 January 2000 to 31 December 2024 were included. The steering committee developed and reviewed the questionnaire using an iterative process.

### Constitution of the voting panel

The voting panel comprised surgeons who had performed ≥200 CRS + HIPEC procedures independently; they were predominantly surgical oncologists responsible for the treatment of patients with digestive and primary peritoneal malignancies, as well as a smaller number of gynaecology oncology surgeons. The members of the voting panel were stratified according to their expertise in performing MI CRS + HIPEC during evaluation of the results. The SurveyMonkey (www.surveymonkey.com) platform was used and the questionnaire was sent via e-mail with a 4-week window in which to respond (extended by 4 weeks in the first round) with fortnightly reminders. All questions within the questionnaire were mandatory and most of the questions were closed questions. Additional comments were solicited if a panellist disagreed or abstained. Two Delphi rounds took place between April and August 2025.

### Achieving a consensus^9^

A response rate of ≥75% was sought in each round. Consensus was reached when >70% of the panellists agreed with any one option, with strong consensus in the case of >90% agreement. If the agreement was <25%, then the question was reframed and re-presented. Questions on which consensus was not achieved in round I were put to a vote in round II. Only two rounds of voting were planned. If consensus was not achieved after two rounds, those questions were to be presented in the results stating that no consensus was achieved.

## Results

For the expert panel, 90 surgeons were invited and 75 (83%) voted in both rounds, of which 97.2% were surgical oncologists and 2.8% were gynaecology oncology surgeons. Only 40% of panellists had experience of ≥10 cases of MI CRS + HIPEC. The overall experience with MI CRS + HIPEC was as follows: no experience, 17.1%; 0–9 cases, 42.8%; 10–50 cases, 30%; and >50 cases, 10%. Only 20% of the panellists had experience with robotic-assisted laparoscopic CRS procedures.

In round I, consensus was reached as follows: SL, 9 of 24 questions (37.5%); RR MI CRS + HIPEC, 0 of 6 questions (0%); MI CRS + HIPEC, 10 of 22 questions (45.4%); and limitations of MIS, 3 of 4 questions (75%). In round II, consensus was reached as follows: SL, 5 of 15 questions (33.3%); RR MI CRS + HIPEC, 2 of 6 questions (33.3%); MI CRS + HIPEC, 6 of 12 questions (50%); and limitations of MIS, 0 of 1 question (0%). Final consensus was reached on 35 of 56 questions (62.5%): SL, 14 of 24 questions (58.3%); RR MI CRS + HIPEC, 2 of 6 questions (33.3%); MI CRS + HIPEC, 16 of 22 questions (72.7%); and limitations of MIS, 3 of 4 questions (75%).

### SL (*Table 1* and *Table S1*)

SL was strongly recommended in the work-up of patients with known PM (agree: 92.7%). For patients with a radiological suspicion of PM, SL was strongly recommended for diagnosis and staging (agree: 81.1%). The panel recommended that the goals of SL should be: evaluation of resectability and/or estimation of the PCI (agree: 78.2%). Previous surgical procedures were not considered a contraindication to SL (agree: 78.2%). A PM referral centre was considered the preferred choice for performing SL and, where performed at non-specialist centres, a video recording was recommended to be shared with the referral centre (agree: 85%). Most panellists recommended recording a video during SL (agree: 69%). SL could be performed regardless of the timing of systemic chemotherapy (agree: 82%). Amongst the panellists, SL is performed for all patients with PM by 40.5%, after neoadjuvant therapy to evaluate resectability by 13.0%, and in borderline resectable cases only by 44.9%. A complete SL includes exploration of all 13 regions (agree: 80%).

**Table 1:** Consensus recommendations for SL

Recommendations	Percentage of votes
In the work-up of patients with a clinical suspicion of peritoneal carcinomatosis, the use of SL must be strongly recommended in selected patients.	81.16
SL is divided into two different procedures: evaluation of resectability and calculation of the PCI.	78.26
SL must be done by the PM referral centre, otherwise a video must be recorded and sent.	85.51
The SL could be performed at any point during chemotherapy and there is no need to disrupt the planned cycles.	82.61
No ligaments should be divided during SL.	81.16
Ports (12 mm) must be closed by suture; even more so in the presence of ascites.	92.75
Previous surgeries are not a contraindication to SL.	78.26
In PMP, SL is not needed systematically, only for selected cases.	71.01
In gastric peritoneal metastases, SL must be performed before any treatment if there is any chance of resectability.	86.96
The optical scope must be angulated (30°, 45°, or flexible).	88.41
SL must visualize all 13 regions in order to assess the completeness, quality, and adequacy	80.60

SL, staging laparoscopy; PM, peritoneal malignancy; PCI, peritoneal cancer index; PMP, pseudomyxoma peritonei.

The patient must be secured to the operating table to allow repositioning for the evaluation of occult peritoneal spaces (agree: 73.9%) and the optical scope used must be angulated (agree: 88.4%). Port sites (12 mm) must by sutured in layers, especially in the presence of ascites (agree: 92.7%). In the evaluation of the PCI, hidden regions or occult peritoneal spaces that should be routinely inspected are: the right subphrenic region (agree: 78.6%), the lesser omentum (agree: 75.3%), and the small bowel and its mesentery (agree: 89.8%). Peritoneal ligaments should not be divided systematically to explore the peritoneal cavity (agree: 81.1%). A consensus could not be reached on the placement of ports in the midline (agree: 68%) and the number of ports that should be used (66% of panellists recommended using 3 ports). A consensus was not reached on sampling of ascitic fluid or performing peritoneal washings when peritoneal nodules are present. No consensus was reached on the routine resection of port sites during subsequent CRS and no prior surgical score (PSS) was considered a contraindication to SL.

The indications for SL stratified by tumour origin are: gastric peritoneal metastases (SL was recommended before any treatment was commenced in patients who had the potential to achieve complete resection (agree: 86.9%)), pseudomyxoma peritonei (PMP) (though no consensus was reached, the majority of the panel recommended that SL is not needed for all patients (agree: 71.0%)), advanced ovarian cancer (no consensus was reached on performing SL either before or after neoadjuvant treatment (agree: 57% and 51% respectively), although >50% recommended both procedures), and colorectal peritoneal metastases (routine SL was not recommended (agree: 40%)). In cases of high-grade PM, the use of SL before and after neoadjuvant chemotherapy did not reach consensus, although the percentage of panellists recommending it was high (agree: 68%).

### RR MI CRS + HIPEC (*Table 2* and *Table S2*)

RR MI CRS + HIPEC was not recommended for all patients with LAMN type II or locally advanced colon cancer (no consensus reached). For LAMN type II lesions there was no consensus for surgical management over observation; however, if surgery is undertaken it should include resection of the appendiceal stump and right iliac fossa peritoneum, omentectomy, and bilateral oophorectomy in postmenopausal women (agree: 78%).

**Table 2:** Consensus recommendations for RR MI CRS + HIPEC

Recommendations	Percentage of votes
When performing RR MI CRS + HIPEC for LAMN type II, the surgery must include: appendiceal stump, right iliac fosse peritoneum, omentum, and bilateral oophorectomy in postmenopausal women.	77.94
When performing RR MI CRS + HIPEC for high-risk colon cancer, the surgery must include: primary tumour plus involved organs and target surgery (omentum, round ligament, appendix, and bilateral oophorectomy in postmenopausal women).	82.35

RR, risk-reducing; MI, minimally invasive; CRS, cytoreductive surgery; HIPEC, hyperthermic intraperitoneal chemotherapy; LAMN, low-grade appendiceal mucinous neoplasm.

In patients with cT4 or perforated colon cancer, no consensus was reached for RR MI CRS + HIPEC (agree: 39% and disagree: 27%); however, if surgery is undertaken it should include resection of the omentum, round ligament, and appendix, and bilateral oophorectomy in postmenopausal women (agree: 82%).

### MI CRS + HIPEC (*Table 3* and *Table S3*)

An MI CRS + HIPEC programme has been commenced by 61.7% of respondents. To start such a programme, mentoring was considered essential (agree: 81%). The MI CRS + HIPEC approach was considered to currently be in the ‘Exploration’ phase of the Idea, Development, Exploration, Assessment, Long-term study (IDEAL) framework (agree: 78%). There was no consensus about the minimum number of complex MI oncological procedures that a surgeon should have performed (45.5% recommended ≥30 procedures). MI CRS + HIPEC could be considered an option in patients with low-volume and low-grade PM (agree: 76%); however, no consensus was achieved about patients with high-grade disease (agree: 48%). MI CRS should be contraindicated if SL is unable to assess all regions adequately (agree: 88%). A small midline laparotomy performed before commencing MI CRS to assess the disease on the small bowel could be used for selected patients (agree: 85%).

**Table 3:** Consensus recommendations for MI CRS + HIPEC

Recommendations	Percentage of votes
In the IDEAL framework, the MI CRS + HIPEC technique is allocated to the Exploration phase.	77.94
Phase III clinical trials comparing MI CRS + HIPEC and open CRS are needed.	76.47
MI CRS + HIPEC is considered an option in patients with low-burden and low-grade peritoneal carcinomatosis.	76.81
A PCI evaluation laparoscopy must be performed before considering to continue using MI CRS + HIPEC. This must explore all the abdominal regions, spending the necessary time.	91.18
MI CRS should be contraindicated if SL is unable to assess all regions adequately.	88.41
HIPEC should be administered through the ports.	75.00
The patient must be secured to the operating table to ensure correct exploration before deciding to continue using a laparoscopic approach.	85.29
Peritonectomy procedures must be performed during MI CRS + HIPEC in the same way as performed during open surgery.	82.35
A mini midline laparotomy is not necessary for all patients - only for selected cases - before commencing MI CRS + HIPEC to assess the disease on the small bowel.	85.07
Inspection and palpation of the small bowel and its mesentery through a mini-laparotomy during MI CRS + HIPEC should be done only for selected cases and not systematically.	76.60
The preferred route of extraction of specimens after MI CRS + HIPEC with endo-bag is the mini-laparotomy.	79.41

MI, minimally invasive; CRS, cytoreductive surgery; HIPEC, hyperthermic intraperitoneal chemotherapy; IDEAL, Idea, Development, Exploration, Assessment, Long-term study; PCI, peritoneal cancer index; SL, staging laparoscopy.

**Fig. 1:**
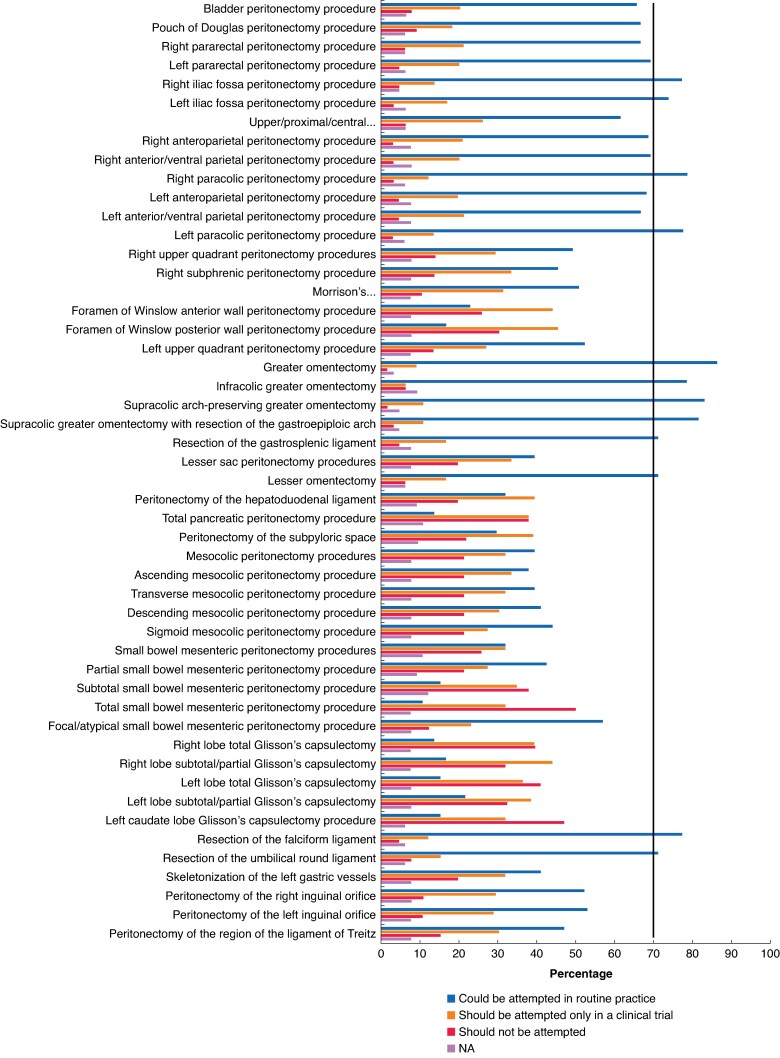
Peritonectomy procedures defined by the Lyon consensus that may be performed using an MI approach in routine practice MI, minimally invasive; NA, not answer.

The panellists recommended that right and left paracolic peritonectomy procedures, greater omentectomy, infracolic greater omentectomy, supracolic arch-preserving greater omentectomy, supracolic greater omentectomy with resection of the gastroepiploic arch, resection of the gastrosplenic ligament, lesser omentectomy, resection of the falciform ligament, and resection of the umbilical ligament could be performed during MI CRS, according to the boundaries defined by the Lyon consensus (*Fig. 1*). No consensus was reached on performing other procedures.

There was no consensus about the ideal port placement for MI CRS (*Table S3*). HIPEC should be administered through the MI CRS port sites (agree: 75%). As for SL, patients should be appropriately secured to the operating table (agree: 85%). Of the respondents, 91% considered laparoscopic estimation of the PCI essential before commencing MI CRS ± HIPEC. The panel recommended that peritonectomy procedures should be performed in the same manner as the open approach (agree: 82%). The preferred route of extraction of specimens placed in an endo-bag was a mini-laparotomy (agree: 79%).

**Fig. 2:**
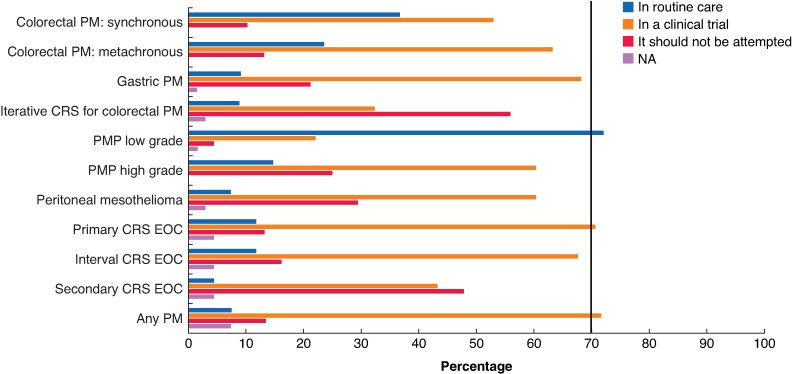
Indications for performing MI CRS ± HIPEC according to tumour type, timing of peritoneal metastases, and tumour grade MI, minimally invasive; CRS, cytoreductive surgery; HIPEC, hyperthermic intraperitoneal chemotherapy; PM, peritoneal malignancy; PMP, pseudomyxoma peritonei; EOC, ephitelial ovarian cancer; NA, not answer.

The MI approach was considered acceptable only for patients with low-grade PMP and a low PCI. For all other types of PM, no consensus was reached. The option that received the most votes was performing such procedures in a clinical trial (*Fig. 2*). Phase III clinical trials to compare the MI and open approaches were recommended (agree: 76%), with a non-inferiority design (agree: 77%). No consensus was reached on an appropriate primary endpoint for such trials, though the majority favoured peritoneal progression-free survival (agree: 63%).

### Limitations of MIS

SL was thought to risk underestimation of the PCI by 94% of the panellists, the main reasons being inexperience of the surgeon, previous surgery, or disease in inaccessible regions (*Table 3*). Technical limitations of MI CRS ± HIPEC were considered to be: a lack of tactile sensation (69%), an inability to explore and assess ‘hidden regions’ (83%), and adhesions caused by a tumour and/or previous surgery (82%).

The limit of tumour burden for MI CRS + HIPEC was considered to be a PCI of ≤10 (agree: 60.2%), with 35% recommending an MI approach only for patients with a PCI <5. Though no consensus was reached, the panel recommended a more restrictive PCI (<5) for high-grade tumours (agree: 69.5%). The involvement of more than two regions was considered a contraindication by 82% of panellists and the involvement of more than one region was considered a contraindication by 17.6% of panellists.

## Discussion

This is the first consensus addressing the controversial issue of MIS for PM. There was a high participation rate of ≥75% in both rounds with a consensus reached on 62.5% of the questions. Just over a decade ago, the first reports on CRS using an MI approach were published, which challenged the conventional norm that these complex procedures could only be performed using the traditional xiphoid-pubis incision. Since then, interest and evidence regarding MI approaches has been growing^3,6^. While early reports showed a benefit in terms of faster recovery times with non-inferior early oncological outcomes, long-term safety has not yet been established. Thus, this consensus was essential to define appropriate indications and provide recommendations on the technical considerations of MI CRS and future research in the field.

The value of SL was underlined by this consensus. It was considered useful in the work-up of patients with PM by 91% of the panel, which is in contrast to a 2008 consensus where it was recommended by only 10% of experts^10^. SL not only avoids futile laparotomy and predicts the likelihood of a complete resection in patients with advanced ovarian cancer, it also helps in evaluating the PCI and studying the sites of disease to aid surgical planning^11,12^. Consensus was achieved on some technical aspects, including the use of an angulated scope, securing the patient to the operating table, and video recording of the procedure. An important recommendation was to perform these procedures only at specialized centres. A poor concordance between laparoscopic and laparotomy findings was shown when SL is performed by non-specialist surgeons^13^. Although consensus was strongly achieved for the use of SL, it was not specifically achieved for colorectal and ovarian cancer patients. This is an emerging concept, as changes in the PCI after neoadjuvant treatment may provide valuable information about the response to systemic chemotherapy and facilitate optimal selection of patients for CRS^14^.

RR MI CRS + HIPEC (also known as prophylactic MI CRS + HIPEC) remains a controversial intervention. Several groups have reported good outcomes for LAMN with mucin extravasation or rupture during initial surgery (LAMN type II), colonic adenocarcinoma with high risk of peritoneal dissemination or locoregional recurrence (T4 and perforated tumours; ovarian metastasis), and locally advanced gastric cancer without peritoneal metastases^15–19^. Definitive evidence showing a clear survival benefit is lacking, hence no consensus was reached on five of six questions in this section.

Regarding MI CRS ± HIPEC, the panel was very restrictive in its recommendations. Low-grade PMP with low-volume disease was considered the only indication for performing MI CRS + HIPEC in routine practice, with a recommendation that it is only used for other indications within the setting of a clinical trial. MI CRS should be performed as per the open procedure, with a non-inferiority phase III randomized trial undertaken to establish the safety of the procedure^20,21^.

The main limitation of this consensus exercise is that it is underpinned by low levels of evidence, possible under-representation of surgeons currently performing MIS, and under-representation of gynaecology oncology surgeons. However, this consensus should be considered the starting point for bringing about uniformity in the practice of those who are performing MIS for PM and to guide surgeons who wish to adopt and investigate this approach in the future.

## Collaborators

Alexander Heriot (Peter MacCAllum Cancer Centre, Melbourne, Australia); Almog Ben-Yaacov (Chain Sheba Medical Centre, Ramat-Gan, Israel); Andrea Di Giorgio (Fondazione Policlinico Universitario A Gemelli, IRCCS, Rome, Italy); Andreas Brandl (University Hospital Heidelberg, Heidelberg, Germany); Andrew M. Lowy (UC San Diego, La Jolla, USA); Armando Sardi (Mercy Medical Center, Baltimore, USA); Avanish Saklani (Tata Memorial Hospital, Mumbai, India); Beate Rau (Charité Hospital, Berlin, Germany); Brian Badgwell (MD Anderson Cancer Center, Houston, USA); Claramae Chia (National Cancer Centre, Singapore); Dario Baratti (National Cancer Institute, Milan, Italy); Dileep Damodaran (MVR Cancer Centre and Research Institute, Kozhikode, India); Edward Levine (Wake Forest University, Winston-Salem, USA); Emel Canbay (Private Hospital, Istanbul, Turkey); Clarisse Eveno (Lille University Hospital, Lille, France); Faheez Mohamed (PMI Basingstoke, Basingstoke, UK); Fernando Pereira (Hospital Universitario de Fuenlabrada, Madrid, Spain); Gabriel Glockzin (München Klinik Bogenhausen, Munich, Germany); Diane Goere (Saint-Louis, Paris, France); Ignace De Hingh (Catharina Cancer Institute, Eindhoven, The Netherlands); Jeremiah Deneve (University of North Carolina, Chapel Hill, USA); Jesus Esquivel (Beebe Healthcare, Lewes, USA); Jula Veerapong (UC San Diego, La Jolla, USA); Konstantinos Votanopoulos (Wake Forest University, Winston Salem, USA); Lana Bijelic (Complex Hospitalari Universitari Moises Broggi, Barcelona, Spain); Laura Lambert (Huntsman Cancer Institute, Salt Lake City, USA); Lucas Sideris (University of Montreal, Montreal, Canada); Luis Gonazalez Bayon (Hospital Gregorio Marañón, Madrid, Spain); M. Haroon A. Choudry (University of Pittsburgh Medical Center, Pittsburgh, USA); Marcello Guaglio (Fondazione IRCCS Istituto Nazionale dei Tumori, Milan, Italy); Frederich Marchal (Institut de Cancérologie de Lorraine, Vandoeuvre les Nancy, France); Martin Hübner (CHUV Lausanne, Lausanne, Switzerland); Michele De Simone (FPO, Turin, Italy); Nabila Ansari (Royal Prince Alfred Hospital, Sydney, Australia); Nayef Alzahrani (King Abdulaziz Medical City, Riyadh, Saudi Arabia); Niels Kok (The Netherlands Cancer Institute, Amsterdam, The Netherlands); Oliver Eng (University of California, Irvine, Orange, USA); Olivia Sgarbura (Institut du Cancer Montpellier, Montpellier, France); Paolo Sammartino (Sapienza University, Rome, Italy); Pedro Antonio Cascales Campos (Hospital Clinico Universitario Virgen De La Arrixaca, Murcia, Spain); Pedro Bretcha-Boix (Hospital Quironsalud Torrevieja, Torrevieja, Spain); Peter Cashin (Uppsala University Hospital, Uppsala, Sweden); Marc Pocard (Université Paris Cité, Paris, France); Pompiliu Piso (Hospital Barmherzige Brüder, Regensburg, Germany); Ramakrishnan Ayaloor Seshadri (Cancer Institute (WIA), Chennai, India); Robert M. Barone (UC San Diego, La Jolla, USA); Roman Yarema (Danylo Halytsky Lviv National Medical University, Lviv, Ukraine); Ruiqing Ma (Aerospace Centre Hospital, Beijing, China); Sanjeev Dayal (Peritoneal Malignancy Institute, Basingstoke, UK); Sanket Mehta (Specialty Surgical Oncology Hospital, Mumbai, India); Santiago González-Moreno (MD Andersen Cancer Center, Madrid, Spain); Satish Warrier (Peter MacCallum Cancer Centre, Melbourne, Australia); Sean Dineen (Moffitt Cancer Center, Tampa, USA); Selman Sokmen (Dokuz Eylül University Hospital, Izmir, Turkey); Sherif Abdel-Misih (Stony Brook University Hospital, Stony Brook, USA); Spiliotis John (European Interbalkan Medical Centre, Thessaloniki, Greece); Abdelkader Taibi (Digestive Department, Dupuytren University Hospital, Limoges, France); Tom Cecil (Peritoneal Malignancy Institute, Basingstoke, UK); Vadim Gushchin (Mercy Medical Center, Baltimore, USA); Victor Verwaal (SUS Malmö, Malmö, Sweden); Willemien van Driel (The Netherlands Cancer Institute, Amsterdam, The Netherlands); Wim Ceelen (Ghent University Hospital, Ghent, Belgium); Yan Li (Beijing Tsinghua Changgung Hospital, Beijing, China); Yanghee Woo (City of Hope Comprehensive Cancer Center, Duarte, USA); Yonemura Yutaka (Kishiwada Tokusukai Hospital, Kishiwada, Japan); Zoltan Novak (National Institute of Oncology, Budapest, Hungary); Naoual Bakrin (Lyon University Hospital, Lyon, France); Joel Baumgartner (Moores Cancer Center, San Diego, USA); Cecile Brigand (Strasbourg University Hospital, Strasbourg, France); Jeremiah Deneve (University of Tennessee Health Science Center, Memphis, USA); Marcello Deraco (Istituto Nazionale dei Tumori Milano, Milan, Italy); Pierre Dube (Montreal University Montreal, Canada); Vahan Kepenekian (Lyon University Hospital, Lyon, France); Brian Loggie (Creighton University Medical Center, Omaha, USA); Andrew Lowy (UC San Diego, La Jolla, USA); Brendan Moran (Peritoneal Malignancy Institute, Basingstoke, UK); David Morris (University of New South Wales, Sydney, Australia); Claudio Quadros (Hospital São Rafael, Salvador, Brazil); Francois Quenet (Montpellier Cancer Institute, Montpellier, France); Paul Sugarbaker (Washington, USA); Antonio Apostolos Tentes (Didymoteicho General Hospital / Metropolitan Hospital, Didymoteicho, Greece); Melissa Teo (Singapore General Hospital, Singapore); Kiran Turaga (University of Chicago Medicine, Chicago, USA); Snita Sinukumar (Jehangir Hospital, Pune, India); Marc Lopez (Philippine General Hospital, The Philippines); Amine Souadka (National Cancer Centre, Rabat, Morocco); Rafael Seitenfuis (Clínica Seitenfus, Brazil).

## Supplementary Material

znag019_Supplementary_Data

## Data Availability

The data that support the findings of this study are available on request from the corresponding author. The data are not publicly available due to privacy or ethical restrictions.
