# Supplementary material for: PSOGI consensus on minimally invasive surgery for peritoneal surface malignancy
Source: Br J Surg. 2026 Mar 4;113(3):znag019. doi: 10.1093/bjs/znag019 (PMC13016804; doi:10.1093/bjs/znag019)
Supplement: znag019_Supplementary_Data [file znag019_supplementary_data.docx]

**Title: The PSOGI consensus on minimally-invasive surgery for peritoneal surface malignancy**

***Authors:*** *Arjona-Sanchez A^1^, Bhatt A^2^*,* *Kazi M^3^, Somashekar SP^4^, Raoof M^5^, Cortés D^6^, Kusamura S^7^., Van der Speeten k^8^., Sommariva A^9^., Alyami M.^10^ and Glehen O^11,12^ and PSOGI collaborators.*

**: Author considered as co-first author.*

*1.- Unit of Oncologic and Pancreatic Surgery, University Hospital Reina Sofía and Maimónides Biomedical Research Institute of Córdoba (IMIBIC)/Reina Sofia University Hospital/ University of Córdoba, Spain*

*2.- Department of Surgical Oncology, Shalby Cancer and Research Institute, Ahmedabad, India.*

*3.- Department of Surgical Oncology, Tata Memorial Hospital, Homi Bhabha National Institute, Mumbai, Maharashtra, India.*

*4.- Department of Surgical Oncology, Aster International Institute of Oncology, Bangalore, India*

*5.- Department of Surgery, City of Hope National Medical Center, Duarte, CA, USA.*

*6.- Department Surgical Oncology IVOMED, Madrid Spain.*

*7.- Department of Surgical Oncology, Fondazione IRCCS Istituto Nazionale dei Tumori, Milan, Italy.*

*8.- Department of Surgical Oncology, Zeikenhuis Oost-Limberg, Genk, Belgium.*

*9.- Surgical Oncology of Digestive Tract, Veneto Institute of Oncology IOV-IRCCS, Padua, Italy.*

*10.- Department of Surgery, Oncology center, King Khalid hospital, Najran, Saudi Arabia.*

*11.- Department of Surgical Oncology, Centre Hospitalier Lyon-sud, Lyon, France.*

*12.-CICLY, Lyon 1 University, Lyon, France*

**Corresponding author:** Arjona-Sanchez A. Unit of Oncologic and Pancreatic Surgery, University Hospital Reina Sofía and Maimónides Biomedical Research Institute of Córdoba (IMIBIC)/Reina Sofia University Hospital/ University of Córdoba, Spain. Email: [alvaroarjona@hotmail.com](mailto:alvaroarjona@hotmail.com). Address: Menendez Pidal Av s/n 14004, Cordoba, Spain.

**Supplementary Materials**

Table 1. Recommendations for performing staging laparoscopy after two rounds of voting.

Table 2 Recommendations of risk-reducing cytoreductive surgery and HIPEC after two rounds of voting.

Table 3: Recommendations for minimally-invasive cytoreductive surgery and HIPEC (MI-CRS+ HIPEC) after two rounds of voting.

| Table 1. Recommendations for performing staging laparoscopy after two rounds of voting.   \| **Questions** \| **Options** \| **Round I** \| **Round II** \| **Consensus** \| \| --- \| --- \| --- \| --- \| --- \| \| In the work-up of patients with suspicious of PC the use of SL must be strongly recommended. \| - Agree in all patients to be selected for CRS and HIPEC - Agree only for selected patients - Disagree it is not necessary - Other (please specify) \| 27.54%  65.22%  0.00%  7.25% \| 17.39%  81.16%  0.00%  1.45% \| Reached \| \| SL could be divided in two different procedures: i) resectability evaluation SL or ii) PCI calculation SL for established PC \| - Agree - Neither agree nor disagree - Disagree - Other (please specify) \| 66.67%  13.04%  14.49%  5.80% \| 78.26%  8.70%  8.70%  4.35% \| Reached \| \| In case of neoadjuvant therapy (mostly in high grade tumours), SL should be done before and after neoadjuvant chemo \| - Agree - Neither agree nor disagree - Disagree \| 58.82%  25.00%  16.18% \| 68.12%  18.84%  13.04% \| Not reached \| \| For SL the allocation of ports must be in middle line. \| - Agree - Neither agree nor disagree - Disagree \| 62.82%  24.29%  12.86% \| 68.12%  21.74%  10.14% \| Not reached \| \| How many ports are considered for SL? \| - 2 - 3 - 4 \| 44.93%  55.07%  0 \| 33.33%  66.67%  0 \| Not reached \| \| We should take sample for ascites or peritoneal washing in presence of peritoneal nodules? \| - Agree - Neither agree nor disagree - Disagree \| 50.72%  21.74%  27.54% \| 55.07%  23.19%  21.74% \| Not reached \| \| SL must be done by the PSM referral centre, in contrary, a video must be recorded and sent. \| - Agree - Neither agree nor disagree - Disagree - Other (please specify) \| 66.67%  11.59%  14.49%  7.25% \| 85.51%  2.90%  5.80%  5.80% \| Reached \| \| Ports from a previous SL must be removed during the CRS+HIPEC \| - Agree - Neither agree nor disagree - Disagree - Other (please specify) \| 53.62%  23.19%  15.94%  7.25% \| 55.07%  28.99%  14.49%  1.45% \| Not reached \| \| Which Previous Surgical Score would you establish as contraindication for SL? \| - 0 - 1 - 2 - 3 - Never is a contraindication - Other (please specify) \| 0  0  17.65%  27.94%  36.76%  17.65% \| 0.00%  0.00%  2.90%  26.09%  65.22%  5.80% \| Not reached \| \| For primary ovarian peritoneal metastases, the SL must be performed systematically pre-treatment \| - Agree - Neither agree nor disagree - Disagree - Other (please specify) \| 45.59%  20.59%  26.47%  7.35% \| 57.35%  13.24%  22.06%  7.35% \| Not reached \| \| For primary ovarian peritoneal metastases, the SL must be performed systematically pre-CRS and after neoadjuvant chemo. \| - Agree - Neither agree nor disagree - Disagree - Other (please specify) \| 49.25%  17.91%  25.37%  7.46% \| 51.47%  20.59%  22.06%  5.88% \| Not reached \| \| In colorectal peritoneal metastases, the SL must be performed systematically before any systemic or surgical treatment \| - Agree - Neither agree nor disagree - Disagree - Other (please specify) \| 31.88%  23.19%  37.68%  7.25% \| 40.58%  15.94%  42.03%  1.45% \| Not reached \| \| Should video recording of disease sites be performed during staging laparoscopy \| - Yes for all patients - Only for selected patients - It is not required - Other (please specify) \| 59.42%  27.54%  8.70%  4.35% \| 69.57%  23.19%  2.90%  4.35% \| Not reached \| \| The SL could be performed during any time of chemotherapy regimen and there is no need to disrupt the planned cycles \| - Agree - Neither agree nor disagree - Disagree - Other (please specify) \| 56.52%  13.04%  18.84%  11.59% \| 82.61%  1.45%  13.04%  2.90% \| Reached \| \| Should adhesiolysis be performed during staging laparoscopy \| - Tumor adhesions should not be divided - Benign adhesions should be completely divided - No adhesiolysis should be performed - Other (please specify) \| 19.12%  27.94%  23.53%  29.41% \| 11.59%  24.64%  34.78%  28.99% \| Not reached \| \| Should any peritoneal ligaments be divided during staging laparoscopy \| - Hepatogastric ligament - Falciform ligament - Right coronary ligament - Left coronary ligament - No ligaments should be divided - Other (please specify) \| 7.25%  5.80%  7.25%  4.35%  81.16%  8.70% \|  \| Reached \| \| Ports (12mm) must be closed by suture, even more in presence of ascites. \| - Agree - Neither agree nor disagree - Disagree - Other (please specify) \| 92.75%  5.80%  0.00%  1.45% \|  \| Strong consensus \| \| Previous surgeries is a contraindication to do SL. \| - Agree - Neither agree nor disagree - Disagree - Other (please specify) \| 4.35%  14.49%  78.26%  2.90% \|  \| Reached \| \| In Pseudomyxoma peritonei SL is not needed systematically. \| - Agree - Neither agree nor disagree - Disagree - Other (please specify) \| 71.01%  11.59%  8.70%  8.70% \|  \| Reached \| \| In gastric peritoneal metastases SL must be performed before any treatment if there is any chance of resectability. \| - Agree - Neither agree nor disagree - Disagree - Other (please specify) \| 86.96%  4.35%  4.35%  4.35% \|  \| Reached \| \| The optical must be angulated (30º,45º or flexible) \| - Agree - Disagree - Other (please specify) \| 88.41%  4.35%  7.25% \|  \| Reached \|   SL: Staging laparoscopy, PCI: peritoneal cancer index.  Table 2 Recommendations of risk-reducing cytoreductive surgery and HIPEC after two rounds of voting.   \| Question \| Options \| Round I \| Round II \| Consensus \| \| --- \| --- \| --- \| --- \| --- \| \| Should RR-CRS-HIPEC be performed by MIS? \| Yes for all histologies (Local advanced colon cancer and LAMN type II) \| 29.85% \| 26.87% \| Not reached \| \| Only for low-risk histology \| 29.85% \| 40.30% \| \| Never should be performed \| 4.48% \| 7.46% \| \| Other (please specify) \| 35.82% \| 25.37% \| \| Low Appendiceal Mucinous Neoplasm type II (presence of mucin outside the appendix serosa) must be treated by RR-MI-CRS+HIPEC instead of observation \| Agree \| 36.76% \| 41.18% \| Not reached \| \| Neither agree nor disagree \| 22.06% \| 11.76% \| \| Disagree \| 29.41% \| 42.65% \| \| Other (please specify) \| 11.76% \| 4.41% \| \| In case of perform RR-MI-CRS+HIPEC in LAMN type II, the surgery must include: appendiceal bump, right iliac fosse peritoneum, omentum and bilateral oophorectomy in post-menopausal women \| Agree \| 66.67% \| 77.94% \| Reached \| \| Neither agree nor disagree \| 5.80% \| 0.00% \| \| Disagree \| 17.39% \| 16.18% \| \| Other (please specify) \| 10.14% \| 5.88% \| \| In high-risk colon cancer (cT4 or perforated) RR-MI-CRS+HIPEC must be offered to these patients. \| Agree \| 39.13% \| 42.65% \| Not reached \| \| Neither agree nor disagree \| 27.54% \| 17.65% \| \| Disagree \| 27.54% \| 38.24% \| \| Other (please specify) \| 5.80% \| 1.47% \| \| In high-risk colon cancer the RR-MI-CRS+HIPEC must include primary tumour plus involved organs and target surgery (omentum, round ligament, appendix, bilateral oophorectomy in postmenopausal women). \| Agree \| 59.42% \| 82.35% \| Reached \| \| Neither agree nor disagree \| 18.84% \| 1.47% \| \| Disagree \| 15.94% \| 13.24% \| \| Other (please specify) \| 5.80% \| 2.94% \| \| RR-MI-CRS+HIPEC could be offered in locally advanced and/or positive cytology gastric cancer after neoadjuvant therapy. \| Agree \| 53.62% \| 69.12% \| Not reached \| \| Neither agree nor disagree \| 21.74% \| 8.82% \| \| Disagree \| 20.29% \| 20.59% \| \| Other (please specify) \| 4.35% \| 1.47% \|   Table 3: Recommendations for minimally-invasive cytoreductive surgery and HIPEC (MI-CRS+ HIPEC) after two rounds of voting.   \| Question \| Options \| Round 1 \| Round 2 \| Consensus \| \| --- \| --- \| --- \| --- \| --- \| \| To start a MI CRS+ HIPEC programme, what should be minimum number of advanced MI oncological resection performed by the surgeon? \| - 10-30 - 30-50 - 50-70 - > 70 \| 16.67%  31.82%  9.09%  42.42% \| 10.29%  45.59%  5.88%  38.24% \| Not reached \| \| Where is MI CRS+ HIPEC in the IDEAL framework of new surgical technologies? \| - Idea - Development - Exploration - Assessment - Long term studies - Other (please specify) \| 10.14%  18.84%  40.58%  18.84%  10.14%  1.45% \| 4.41%  13.24%  77.94%  2.94%  0.00%  1.47% \| Reached \| \| Are phase III clinical trials comparing MI CRS+ HIPEC and open CRS needed ? \| - Yes - No \| 69.57%  30.43% \| 76.47%  23.53% \| Reached \| \| What should be the primary end-points of such trials ? \| - Overall survival - DFS - Peritoneal free survival - perioperative outcomes - Cost effectiveness - Qol/PROMs - Other (please specify) \| 20.59% 25.00%  9.41%  7.35%  1.47%  1.47%  14.71% \| 13.24%  11.76%  63.24%  1.47%  0.00%  1.47%  8.82% \| Not reached \| \| Do you consider MI CRS+ HIPEC as an option in patients with low burden and low-grade PC? \| - Yes - No - Other (please specify) \| 76.81%  14.49%  8.70% \|  \| Reached \| \| Do you consider MI CRS+ HIPEC as an option in patient with low burden and high-grade PC? \| - Yes - No - Other (please specify) \| 48.53%  50.00%  1.47% \| 38.24%  61.76%  0.00% \| Not reached \| \| What PCI would you consider a limitation for MI CRS+ HIPEC for low grade PC? \| - <5 - <10 - ≤15 \| 32.35%  54.41%  4.41% \| 35.29%  60.29%  0.00% \| Not reached \| \| Is the number of regions of peritoneum involved a consideration for MI CRS+ HIPEC? \| - Single region - up to 2 regions - up to 3 regions - Any number of regions \| 18.18%  40.91%  19.70%  21.21% \| 17.65%  64.71%  7.35%  10.29% \| Not reached \| \| A PCI evaluation laparoscopy must be performed before considering continuing by MI CRS+ HIPEC. This must explore all the abdominal regions spending the necessary time \| - Agree - Neither agree nor disagree - Disagree - Other (please specify) \| 91.18%  4.41%  4.41%  0.00% \|  \| Strong consensus \| \| MI-CRS should be contraindicated if staging laparoscopy is unable to assess all regions adequately \| - Agree - Neither agree nor disagree - Disagree - Other (please specify) \| 88.41%  7.25%  2.90%  1.45% \|  \| Reached \| \| How should the completeness/ quality/ adequacy of staging laparoscopy be assessed \| - Surgeons own assessment - All 13 regions should be visualized - A critical structure in each of the 13 regions should be visualized - Other (please specify) \| 33.82%  57.35%  30.88%  8.82% \| 20.90%  80.60%  13.43%  4.48% \| Reached \| \| The allocation of ports must be in transverse middle line to allow you the access to low and upper abdomen. \| - Agree - Neither agree nor disagree - Disagree - Other (please specify) \| 51.47%  29.41%  14.71%  4.41% \| 55.88%  35.29%  7.35%  1.47% \| Not reached \| \| HIPEC could be administered through the ports \| - Agree - Neither agree nor disagree - Disagree - Other (please specify) \| 75.00%  16.18%  7.35%  1.47% \|  \| Reached \| \| The patient must be fixed to the operating table to ensure a correct exploration before deciding to continue by laparoscopic approach. \| - Agree - Neither agree nor disagree - Disagree - Other (please specify) \| 85.29%  13.24%  1.47%  0.00% \|  \| Reached \| \| Peritonectomy procedures must be performed during MI CRS+ HIPEC in the same way as performed during open surgery \| - Agree - Neither agree nor disagree - Disagree - Other (please specify) \| 67.16%  22.39%  8.96%  1.49% \| 82.35%  10.29%  2.94%  4.41% \| Reached \| \| Should a mini midline laparotomy be performed before commencing MI CRS+ HIPEC to assess the disease on the small bowel? \| - In all Patients - In selected patients - Not indicated - Other (please specify) \| 15.94%  55.07%  26.09%  2.90% \| 2.99%  85.07%  8.96%  2.99% \| Reached \| \| Inspection and palpation of the small bowel and its mesentery through a mini-laparotomy during MI CRS+ HIPEC \| - Should be performed for all patients - Could be performed for selected patients (e.g. high-grade disease) - Is not indicated \| 21.28 %  76.60 %  2.13 % \|  \| Reached \| \| What should be the preferred route of delivery of specimens after MI CRS+ HIPEC with endo-bag. \| - Natural orifices if possible - Mini-laparotomy - Other (please specify) \| 14.71%  79.41%  5.88% \|  \| Reached \|   MIS: minimally invasive surgery, GI: gastrointestinal, PC: peritoneal carcinomatosis, PCI: peritoneal cancer index; QoL- quality of life; PROM- patient reported outcome measures |  |
| --- | --- | --- | --- | --- | --- | --- | --- | --- | --- | --- | --- | --- | --- | --- | --- | --- | --- | --- | --- | --- | --- | --- | --- | --- | --- | --- | --- | --- | --- | --- | --- | --- | --- | --- | --- | --- | --- | --- | --- | --- | --- | --- | --- | --- | --- | --- | --- | --- | --- | --- | --- | --- | --- | --- | --- | --- | --- | --- | --- | --- | --- | --- | --- | --- | --- | --- | --- | --- | --- | --- | --- | --- | --- | --- | --- | --- | --- | --- | --- | --- | --- | --- | --- | --- | --- | --- | --- | --- | --- | --- | --- | --- | --- | --- | --- | --- | --- | --- | --- | --- | --- | --- | --- | --- | --- | --- | --- | --- | --- | --- | --- | --- | --- | --- | --- | --- | --- | --- | --- | --- | --- | --- | --- | --- | --- | --- | --- | --- | --- | --- | --- | --- | --- | --- | --- | --- | --- | --- | --- | --- | --- | --- | --- | --- | --- | --- | --- | --- | --- | --- | --- | --- | --- | --- | --- | --- | --- | --- | --- | --- | --- | --- | --- | --- | --- | --- | --- | --- | --- | --- | --- | --- | --- | --- | --- | --- | --- | --- | --- | --- | --- | --- | --- | --- | --- | --- | --- | --- | --- | --- | --- | --- | --- | --- | --- | --- | --- | --- | --- | --- | --- | --- | --- | --- | --- | --- | --- | --- | --- | --- | --- | --- | --- | --- | --- | --- | --- | --- | --- | --- | --- | --- | --- | --- | --- | --- | --- | --- | --- | --- | --- | --- | --- | --- | --- | --- | --- | --- | --- | --- | --- | --- | --- | --- | --- | --- | --- | --- | --- | --- | --- | --- | --- | --- | --- | --- | --- | --- | --- | --- | --- | --- | --- | --- | --- | --- | --- | --- | --- | --- | --- | --- | --- | --- | --- | --- | --- | --- | --- | --- | --- | --- | --- | --- | --- | --- | --- | --- | --- | --- | --- | --- | --- | --- | --- |
